# The oral – systemic link: oral infection/inflammation and the relation to general health

**DOI:** 10.1080/07853890.2021.1896879

**Published:** 2021-09-28

**Authors:** Bjorn Klinge

**Affiliations:** Karolinska Institute in Stockholm, Sweden

## Abstract

Increasing evidence suggests an independent association between periodontitis and a range of comorbidities, among others: cardiovascular disease, type 2 diabetes and rheumatoid arthritis. Shared inflammatory pathways are likely to contribute to this association, but distinct causal mechanisms remain to be defined. Some of these comorbid conditions may improve by periodontal treatment, and a bidirectional relationship may exist, where, for example, treatment of diabetes can improve periodontal status, and successful treatment of periodontitis lowers blood glucose levels (i.e. HbA1c) in the diabetic patient. In this presentation an overview of the evidence linking periodontitis with selected systemic diseases will be discussed. The available evidence for an oral-systemic link calls for increased cooperation between dentists and medical doctors to provide optimal screening, treatment, and prevention of both periodontitis and its comorbidities.

